# Intrathecal administration of PD-1 inhibitor combined with pemetrexed for leptomeningeal metastases from breast cancer: a case report

**DOI:** 10.3389/fimmu.2025.1567324

**Published:** 2025-04-24

**Authors:** Yushan Huang, Guozi Yang, Miaomiao Liu, Panpan Tai, Xiao Chen, Min Liu, Zhenyu Pan

**Affiliations:** Department of Radiation Oncology, the Third People’s Hospital of Huizhou, Guangzhou Medical University, Huizhou, China

**Keywords:** leptomeningeal metastasis, intrathecal administration, PD-1 inhibitor, pemetrexed, breast cancer

## Abstract

Leptomeningeal metastasis (LM) is a fatal complication of malignant tumors with limited treatment options. Finding more effective therapeutic strategies is of significant importance. This case reports an LM patient from breast cancer treated with intrathecal pemetrexed (15 mg) combined with PD-1 inhibitors (40 mg). The pemetrexed dosing regimen included an induction phase (twice weekly for 2 weeks), a consolidation phase (once weekly for 4 weeks), and a maintenance phase (once monthly). The PD-1 inhibitor dosing regimen included an induction phase (once every 2 weeks for 6 weeks) and a maintenance phase (once monthly). The patient showed good tolerance, with no severe adverse events observed, and achieved favorable therapeutic outcomes, including complete resolution of neurological symptoms, negative conversion of cerebrospinal fluid cytology, and significant reduction of imaging-detected lesions. This case provides a new approach to the treatment of LM, suggesting that intrathecal immunotherapy combined with intrathecal chemotherapy may be a safe and effective treatment option, offering valuable insights for future clinical applications.

## 1 Introduction

Leptomeningeal metastasis (LM) is a fatal complication characterized by the invasion of tumor cells into the subarachnoid space and their subsequent dissemination via cerebrospinal fluid, affecting the entire central nervous system (1–4). Approximately 5% of breast cancer patients develop LM (5, 6). For these patients, LM is associated with a poor prognosis and limited treatment options, resulting in unsatisfactory therapeutic outcomes and a median survival of only 4 months (6–8).

Intrathecal chemotherapy represents one of the primary treatment modalities for LM from solid tumors, including breast cancer. In 2017, we conducted clinical trials of intrathecal pemetrexed chemotherapy (9, 10). The study results showed that the response rate to intrathecal pemetrexed treatment was 67.6%, with a disease control rate of 73.5%. The overall survival ranged from 0.3 to 16.6 months, with a median survival time of 5.5 months and a 1-year survival rate of 21.6%. Meanwhile, the toxicity of this regimen is manageable. As a novel intrathecal chemotherapeutic agent, pemetrexed demonstrated favorable therapeutic efficacy in the treatment of leptomeningeal metastases from solid tumors. It also showed clinical efficacy in patients with relapsed or refractory disease who had previously received intrathecal methotrexate (IT-MTX)/cytarabine (Ara-C).

In recent years, PD-1 inhibitors have been widely used in the treatment of various tumors with promising therapeutic outcomes. However, previous studies employing systemic administration of PD-1 inhibitors for the treatment of LM from solid tumors have shown limited efficacy (11, 12). This limitation is largely attributed to the high molecular weight of PD-1 inhibitors, which restricts their permeability across the blood-brain barrier. Notably, previous studies have demonstrated that intrathecal administration of PD-1 inhibitors in patients with melanoma brain metastases can achieve some therapeutic efficacy, with some patients experiencing prolonged survival (13). A previous phase I/II study demonstrated that intrathecal administration of an immunosuppressant (nivolumab, 50mg, q2w) was well-tolerated and showed potential efficacy in LM patients from melanoma (13). The study indicated that the median overall survival (OS) was 4.9 months, with OS rates of 44% and 26% at 26 weeks and 52 weeks, respectively. Notably, four patients achieved an OS of 74 weeks, 115 weeks, 136 weeks, and 143 weeks (i.e., 2.7 years). The study results indicated that intrathecal nivolumab is safe and well-tolerated, with no dose-limiting toxicity (DLT) observed. Moreover, studies have shown that, under intravenous administration, the cerebrospinal fluid (CSF) concentration of most drugs, including PD-1 inhibitors, chemotherapy drugs, and certain targeted therapies, is significantly lower than their serum concentration (3, 14–16). Due to the distinct immune microenvironment of solid tumors compared to malignant melanoma, the response rate to immunotherapy alone is relatively low, often requiring a combination with chemotherapy or other antitumor treatments. Therefore, we combined intrathecal chemotherapy with intrathecal immunotherapy for the treatment of leptomeningeal metastases from solid tumors.

This case report describes a breast cancer patient with LM who received intrathecal administration of the PD-1 inhibitor combined with pemetrexed chemotherapy, along with local radiotherapy for selected intracranial lesions. The patient demonstrated good tolerance to the treatment without observed serious adverse events (AEs). Follow-up examinations revealed significant regression of intracranial lesions, including those not directly treated with radiotherapy, suggesting an abscopal effect. Overall, the treatment achieved favorable therapeutic outcomes.

## 2 Case report

### 2.1 Initial diagnosis and treatment

A 40-year-old female patient was diagnosed with breast cancer in September 2019, and underwent surgical resection following neoadjuvant chemotherapy. Postoperative staging was ypT2N2aM0-IIA (HER2-positive type). She received postoperative adjuvant radiotherapy, chemotherapy (including paclitaxel and capecitabine), and anti-HER2 targeted therapy (trastuzumab, pertuzumab, pyrotinib, T-DM1 [ado-trastuzumab emtansine], trastuzumab deruxtecan [fam-trastuzumab deruxtecan], and lapatinib). In October 2020, brain metastases were detected, and she underwent local radiotherapy. In 2024, the brain metastases were progressive, and she received whole-brain radiotherapy (WBRT) (40 Gy in 20 fractions).

### 2.2 LM diagnosis and treatment

**Figure 1:**
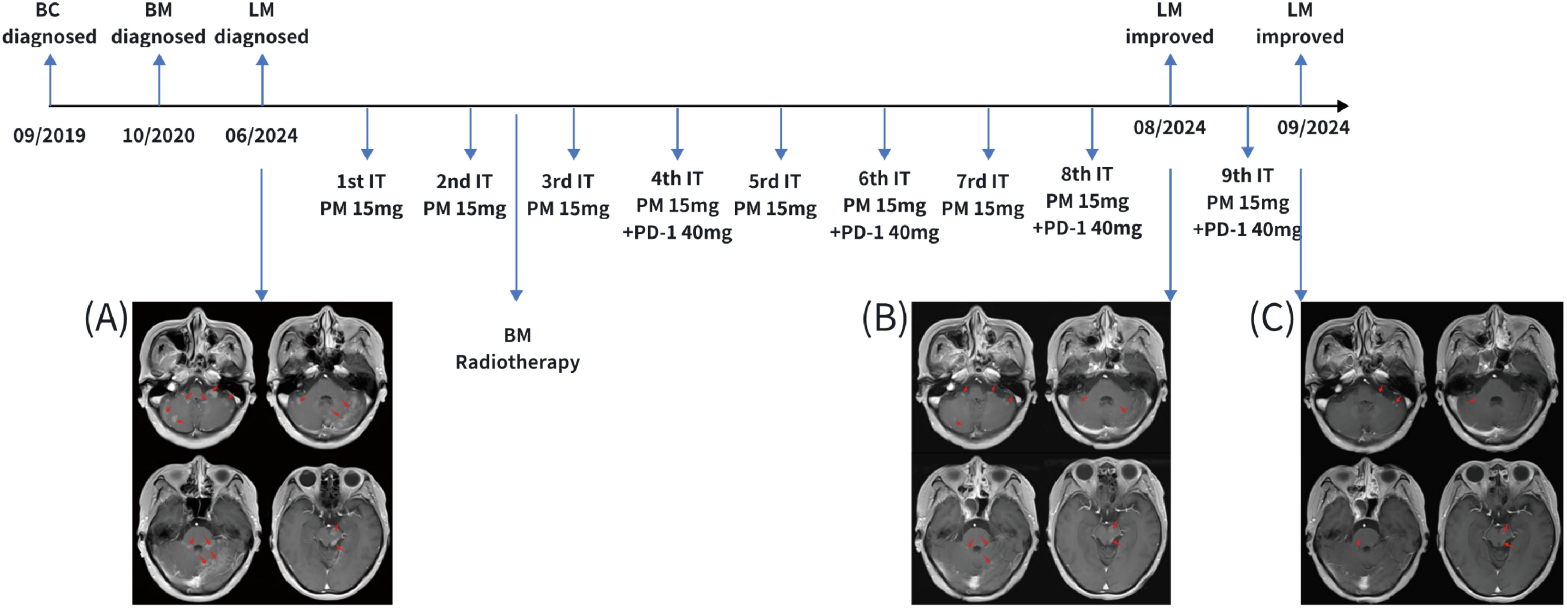
The schedule for intrathecal injection and central imaging. **(A)** Imaging of leptomeningeal metastasis at initial diagnosis in June 2024; **(B)** Imaging after 8 cycles of induction and consolidation therapy with intrathecal injections, showing a significant reduction in the area of leptomeningeal enhancement; **(C)** Imaging after 9 cycles of intrathecal injections, showing further reduction in the area of leptomeningeal enhancement. BC, breast cancer; BM, brain metastasis; LM, leptomeningeal metastasis; IT PM, intrathecal pemetrexed treatment; PD-1, PD-1 inhibitor.

In June 2024, the patient developed neurological symptoms, including reduced sensation in the right side of the head and right upper limb, as well as abnormal gait. Tumor cells were detected in the CSF, confirming the diagnosis of leptomeningeal metastasis. Magnetic resonance imaging (MRI) demonstrated extensive linear and nodular enhancement in the cerebellar sulci, as well as brain parenchymal metastases and nodular lesions (**Figure 1A**).

Subsequently, the patient initiated intrathecal therapy, which comprised the administration of pemetrexed (15 mg) and a PD-1 inhibitor (40 mg). Pemetrexed was administered in three phases: induction phase (twice weekly for 2 consecutive weeks), consolidation phase (once weekly for 4 consecutive weeks), and maintenance phase (once monthly). The PD-1 inhibitor was administered in two phases: the induction phase (every 2 weeks for a total of 6 doses) and the maintenance phase (once monthly). Given the presence of recurrent parenchymal nodular lesions that were refractory to intrathecal pharmacotherapy alone, local radiotherapy was administered to cerebellar and brainstem metastatic lesions. Given the patient’s history of WBRT six months ago, the radiation dose to metastases adjacent to the brainstem was limited to 16 Gy, delivered in 8 fractions of 2 Gy per fraction, to ensure the brainstem dose remained within a safe range. For other brain metastases, a slightly higher single-fraction dose of 28 Gy was administered in 8 fractions of 3.5 Gy per fraction. The small nodular lesions in both temporal lobes were scattered, few in number, and had a diameter of less than 5 mm; therefore, no radiation was administered. The radiotherapy technique employed was a volumetric modulated arc therapy approach.

### 2.3 Treatment response

After completing eight cycles of intrathecal therapy, the patient achieved complete resolution of neurological symptoms. Meanwhile, CSF cytology turned negative. A re-examination of the MRI of the central nervous system revealed a significant reduction in intracranial metastatic lesions. Notably, brain metastases and metastatic nodules that had not received radiotherapy also showed near-complete resolution or marked reduction in size (**Figure 1B**). In September 2024, after the ninth intrathecal treatment, cranial MRI demonstrated a further reduction in meningeal enhancement (**Figure 1C**). In November 2024, although cranial MRI demonstrated slight enlargement of some original brain metastases, neither the imaging findings nor the clinical symptoms met the criteria for disease progression. Therefore, monthly maintenance of intrathecal chemotherapy was continued.

### 2.4 Adverse events

After the third intrathecal injection, the patient developed grade 3 myelosuppression. This was resolved through the administration of granulocyte-stimulating factor, restoring leukocyte and neutrophil counts to normal levels. Following the sixth injection, grade I elevation of hepatic aminotransferases occurred, which normalized after diammonium glycyrrhizinate treatment. The seventh injection resulted in mild radiculitis, which resolved spontaneously. Before the ninth injection, subclinical hyperthyroidism was noted but remained asymptomatic and was monitored. Prior to the eleventh injection, grade II hypothyroidism developed. It was treated with oral levothyroxine 25μg, and it subsequently normalized.

### 2.5 Follow-up

As of February 2025, the patient had survived over eight months since the diagnosis of LM. CSF remained persistently negative. Subsequently, intrathecal therapy was maintained once a month, along with regular DS-8201 (trastuzumab deruxtecan) systemic treatment.

## 3 Discussion

We report the first case of an LM patient from breast cancer treated with intrathecal administration of PD-1 inhibitor combined with pemetrexed. The patient demonstrated good tolerance and safety. The intracranial lesions showed nearly complete regression, resulting in favorable therapeutic outcomes.

The treatment of LM remains a significant challenge for most cancers, including breast cancer, except for certain tumors, such as lung adenocarcinoma, for which small-molecule targeted therapies have proven effective (17). This therapeutic difficulty primarily stems from the blood-cerebrospinal fluid barrier, which restricts the entry of drugs into the CSF through blood circulation (18). Intrathecal chemotherapy, which involves direct drug administration into the subarachnoid space, has emerged as a primary treatment modality. In China, intrathecal pemetrexed has been widely adopted for the treatment of LM.

Unlike melanoma, which is sensitive to immunotherapy, solid tumors, including breast cancer typically require a combination of chemotherapy and immunotherapy for effective treatment. The primary mechanism underlying this combined approach is that chemotherapy alters the tumor microenvironment and induces DNA damage in tumor cells. The destruction of tumor cells leads to the release of tumor-associated antigens, which enhance the visibility of the tumor to the immune system and induce a robust immune response. This enhanced recognition triggers a stronger immune response, ultimately improving the efficacy of immune checkpoint inhibitors (ICIs) (19). Therefore, we attempted to combine intrathecal administration of immunotherapy and chemotherapy drugs. Initially, pemetrexed was administered intrathecally, followed by combined intrathecal administration of pemetrexed and the PD-1 inhibitor every two weeks.

The patient demonstrated good tolerance to the treatment regimen, with no severe AEs observed throughout the therapeutic course. The observed adverse reactions, including myelosuppression, elevation of hepatic aminotransferases (EHA), and radiculitis, were all common side effects associated with intrathecal pemetrexed chemotherapy. During the maintenance phase, the patient developed subclinical hyperthyroidism, which subsequently progressed to hypothyroidism. This sequence is consistent with immune-related primary hypothyroidism (IR-primary hypothyroidism), the most frequent immune-related adverse event occurring in 6-9% of patients receiving anti-PD-1 therapy. Notably, subclinical hyperthyroidism often precedes the development of hypothyroidism, with the majority of cases manifesting within the first three months of treatment initiation (20). The temporal progression and pattern of thyroid dysfunction in this case—transitioning from hyperthyroidism to hypothyroidism—aligned with the typical clinical presentation described in the literature. After receiving targeted treatment, the patient’s hypothyroidism and associated symptoms showed rapid improvement. For immune-related primary hypothyroidism, early identification can be achieved through regular monitoring of thyroid function (TSH, FT3, FT4). Based on the severity of the condition, levothyroxine replacement therapy should be administered, with continuous follow-up to assess treatment efficacy and disease progression.

After receiving treatment with PD-1 inhibitors combined with pemetrexed, the tumor in this patient was well-controlled, with complete resolution of previous neurological symptoms. Imaging examinations showed the disappearance of intracranial lesions, and cerebrospinal fluid (CSF) cytology also turned negative. This may be closely related to the modulatory effects of chemotherapy drugs on the tumor microenvironment, which further enhanced the antitumor activity of immune checkpoint inhibitors (ICIs). First, chemotherapy not only directly kills tumor cells but may also induce immunogenic cell death (ICD) in tumor cells, releasing tumor-associated antigens, attracting the aggregation of dendritic cells (DCs), and subsequently promoting T cell activation (21). At the same time, chemotherapy can promote the increased release of immune-stimulatory factors, enhancing the immune system’s ability to recognize and attack the tumor (22). In addition, chemotherapy can directly kill immunosuppressive cells (such as MDSCs) and promote the infiltration of immune cells into the tumor microenvironment, thereby enhancing the antitumor activity of immune checkpoint inhibitors (ICIs) (23). Although this case demonstrates the positive effects of intrathecal PD-1 inhibitor combined with pemetrexed in the treatment of LM, the broad applicability of this combination therapy still requires further clinical data to support it.

Notably, the untreated parenchymal brain lesions in this patient also showed shrinkage and disappearance. We attributed this to the abscopal effect. Radiotherapy is the most common approach to inducing the abscopal effect by releasing tumor antigens and stimulating a systemic immune response (24). When tumors are irradiated, DNA damage is a potential mechanism for the occurrence of the abscopal effect, which may lead to the release of damage-associated molecular patterns and neoantigens (25). These neoantigens are engulfed by antigen-presenting cells (APCs), which then present them to CD8+ T cells. CD8+ T cells can stimulate a tumor-specific immune response, recognizing and attacking both primary and metastatic tumors (26–28). In addition, the combination of immune checkpoint inhibitors (ICIs) and radiotherapy can enhance the abscopal effect of local antitumor treatment (29). The combination of radiotherapy and ICIs can prolong the induction of cytotoxic T lymphocytes (CTLs) and natural killer (NK) cells, while inhibiting the activity of pro-tumor immune cells, including MDSCs, M2 macrophages, and Tregs. A previous clinical study on ipilimumab combined with radiotherapy for the treatment of advanced melanoma found that 52% of patients experienced the abscopal effect (30).

## 4 Conclusion

In summary, this represents the first case of a solid tumor patient with leptomeningeal metastases treated with combined intrathecal immunotherapy and chemotherapy. The treatment demonstrated good tolerability, safety, and efficacy, warranting further clinical investigation.

## Data Availability

The original contributions presented in the study are included in the article/supplementary material. Further inquiries can be directed to the corresponding author.
